# Oestradiol measurement during fulvestrant treatment for breast cancer

**DOI:** 10.1038/s41416-019-0378-9

**Published:** 2019-01-25

**Authors:** Laura J. Owen, Phillip J. Monaghan, Anne Armstrong, Brian G. Keevil, Claire Higham, Zena Salih, Sacha Howell

**Affiliations:** 1 Biochemistry Department, Wythenshawe Hospital, Wythenshawe, M23 9LT UK; 2 School of Medical Sciences, Faculty of Biology, Medicine and Health, University of Manchester, Manchester Academic Health Science Centre, Manchester, UK; 3 The Christie Pathology Partnership. The Christie, Wilmslow Road, Manchester, M20 4BX UK; 4 University of Manchester, Faculty of Medical and Human Sciences, Institute of Inflammation and Repair, Manchester, UK; 5 Medical Oncology, The Christie, Wilmslow Road, Manchester, M20 4BX UK; 6 Endocrinology, The Christie, Wilmslow Road, Manchester, M20 4BX UK

**Keywords:** Breast cancer, Hormonal therapies

## Abstract

Biochemical evaluation of menopausal status is used to inform treatment decisions, including clinical trial eligibility in women with oestrogen receptor positive breast cancer. However, fulvestrant may interfere with oestradiol immunoassays and confound accurate assessment in this context. We conducted a service evaluation of two immunoassays and an LC-MS/MS assay to determine the extent of the interference. Serum oestradiol levels were analysed by two immunoassays (Siemens Centaur XP and Abbott Architect) and liquid chromatography-tandem mass spectrometry (LC/MS/MS). Immunoassay gave higher serum oestradiol results than LC-MS/MS at low concentrations, with improved analytical sensitivity demonstrated by LC-MS/MS. Cross-reactivity of fulvestrant was observed for each immunoassay. We have shown that two commonly used immunoassays do not demonstrate the required sensitivity or specificity for the measurement of oestradiol in a breast cancer population. For patients receiving fulvestrant, spurious results may be generated that could impact treatment decisions. LC-MS/MS is recommended in this setting.

## Introduction

Accurate assessment of menopausal status is a vital part of the treatment strategy for women with oestrogen receptor (ER) positive breast cancer.^1^ Eligibility for most clinical trials of endocrine therapy requires stringent assessment of menopausal status including serum oestradiol measurement. Most analytical laboratories utilise immunoassays (IA) for this purpose despite oestradiol concentrations being below the detection limit for commercial IAs in most post-menopausal women. Field Safety Notices released in 2016 by suppliers of commercial IAs described cross-reactivity of fulvestrant (a selective ER degrader) in oestradiol IAs. Regulatory bodies including the Food and Drug Administration and Medicines and Healthcare products Regulatory Agency (MHRA) issued medical device alerts urging caution^2^ and the summary of product characteristics (SPC) for fulvestrant were changed to include the statement ‘Due to the structural similarity of fulvestrant and oestradiol, fulvestrant may interfere with antibody based-oestradiol assays and may result in falsely increased levels of oestradiol’.^3^ Despite this, there is a concern that clinical awareness of this issue is limited and therefore the potential risk for inappropriate suppression of ovarian function and clinical trial exclusion remains.

Mass spectrometry, either gas chromatography mass spectrometry (GC-MS)^4^ or liquid chromatography tandem mass spectrometry (LC-MS/MS) has become the gold standard for assay of oestradiol levels in this clinical situation.^5^ The sensitivity of these assays surpasses that of most immunoassays and is typically 10 pmol/L or lower. The combination of chromatography and mass spectrometry increases specificity. Immunoassays can experience interference in the presence of structurally similar compounds and this is a particular problem for steroid hormones due to the large number of circulating steroids and therapeutic steroid-based compounds and their metabolites.

We have recently established a LC-MS/MS oestradiol assay to enable accurate assessment of women receiving aromatase inhibitor therapy.^6^ We conducted a service evaluation into the effect of fulvestrant interference for two IAs used in our hospitals and compared to a highly sensitive and specific LC-MS/MS assay as gold standard to determine the extent of the reported issue.

## Materials and methods

### Assays

All assays in this study are approved and validated for clinical use and were performed according to the manufacturers’ recommendations. The two IAs investigated, Siemens Centaur XP (SC) and Abbott Architect (AA), describe lower limit of quantitation (LLOQ) for oestradiol detection of 70 and 92 pmol/L, respectively. An established LC-MS/MS assay was used as the gold standard with LLOQ of 10 pmol/L.^6^ Certified reference materials were used in the preparation of calibrators for the LC-MS/MS assay and matrix-matched reference materials were used to validate the prepared calibrators to ensure the traceability of the accuracy of this assay. The coefficients of variation (CVs) were <8% for SC, <9.2% for AA, and <5.1% for LC-MS/MS assays over three quality control levels.

### Patient cohorts

Cohort 1 (*n* = 44): Surplus serum from female patients without a cancer diagnosis. These patients had an age range 17–50 years (median 35 years). Serum that had been sent to the laboratory for oestradiol measurement was anonymised and stored at −20 °C prior to analysis. These samples were used to establish the expected method comparison between each IA and LC-MS/MS.

Cohort 2 (*n* = 16): Post-menopausal women with a breast cancer diagnosis but not receiving endocrine therapy. From these patients, 17 samples were received. The median age was 50 years (range 39–59). This cohort is considered the control population.

Cohort 3 (*n* = 10): Women with advanced breast cancer and receiving treatment with fulvestrant at the time of sampling.

Analyse-it for Microsoft Excel was used for all statistical analyses.

## Results

In cohort 1 (patients without a cancer diagnosis), correlations between IA and LC-MS/MS were excellent (Pearson *R*^2^ = 0.98 between SC and LC-MS/MS and *R*^2^ = 0.99 between AA and LC-MSMS). Bland Altman analysis demonstrated a negative bias for AA [−18.6 pmol/L; 95% limits of agreement (LOA) −85.8 to 48.5] (Supplemental Figure 1) and a positive bias for SC vs LC-MS/MS [37.9 pmol/L; 95% LOA −27.7 to 103.5] (Supplemental Figure 2).

In cohort 2 (post-menopausal patients with a cancer diagnosis), serum oestradiol concentrations were detectable (above the assay-specific LLOQ) in 41% of samples by LC-MS/MS, yet only 12% of samples by AA and SC.

**Fig. 1:**
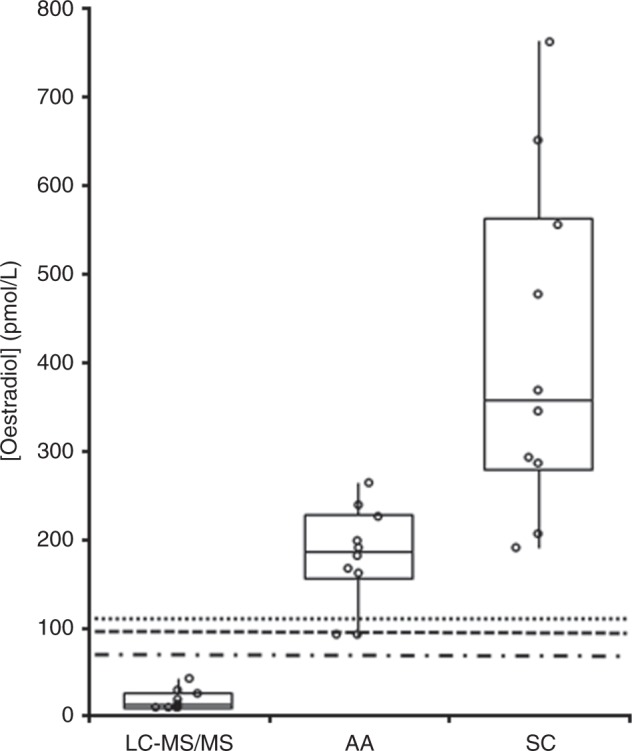
The distribution of results for all three assays for patients receiving fulvestrant. The boxes represent the median, 25th, and 75th centile while the whiskers represent the 95% confidence interval. The upper limit of the post-menopausal range has been highlighted for each assay; the dotted line represents the SC assay (118 pmol/L), the dashed line represents the AA assay (103 pmol/L), and the dashed and dotted line represents the MS assay (77 pmol/L)

In cohort 3 (*n* = 10 patients receiving fulvestrant), oestradiol levels were in the pre-menopausal range for 10/10 by SC, 8/10 for AA, and 0/10 by LC-MS/MS (Fig. 1). Median oestradiol levels were 356 pmol/L (range 191–761) for SC, 186 pmol/L (range <92–263) for AA, and 14 pmol/L (range <10–43) for LC-MS/MS (Mann–Whitney *p* < 0.001 for comparison of both IAs against LS-MS/MS). In two additional patients, oestradiol measurement 2 and 4 months after the last dose of fulvestrant demonstrated elevated levels of 165 and 283 pmol/L by SC and <92 and 124 pmol/L by AA, respectively, with corresponding LS-MS/MS values of 20 and <10, illustrating that the interference persists due to the long half-life of fulvestrant.^7^

## Discussion

In this study we have confirmed the interference of fulvestrant with oestradiol IAs that are used in routine clinical practice. The issue of cross-reactivity between fulvestrant with commercially available IAs was raised in a 2015 case report.^8^ Field Safety Notices, regulatory body alerts, and changes in the SPC followed but this has not alerted practising oncologists to this critically important issue. A spuriously raised oestradiol level may result in inappropriate investigation of ovarian reserve, ovarian function suppression, or exclusion from clinical trial entry with menopausal status as an eligibility criterion. Notably, fulvestrant is administered intramuscularly and results in slow absorption and a half-life at both 250 and 500 mg doses of 54 days.^7^ This protracted decline in circulating fulvestrant levels makes errors in oestradiol assay interpretation more likely as evidenced by our patients with spuriously elevated levels 2 and 4 months after their last dose.

The use of mass spectrometry assays was recommended for oestradiol estimation in women receiving fulvestrant by the MHRA and the data presented in this study support this notion. Furthermore, the comparison of both IAs with LC-MS/MS in a normal population showed that both overestimated oestradiol in the lower concentration range. It is this low concentration range that is of interest when making decisions regarding menopausal status. Although immunoassays are widely available and suitable for use in many patient populations, such as pre-menopausal women, they are limited in the breast cancer population due to their limited sensitivity and specificity. These limitations need to be understood and balanced with the longer turnaround time of a more specific and sensitive mass spectrometric analysis. The mass spectrometry equipment is expensive and oestradiol is a difficult analyte to measure; therefore, the analysis should be limited to centres with this equipment and expertise using a fully validated assay such as this one. Mass spectrometry assays are readily accessible to clinicians and laboratories in the UK, with samples simply referred by first class post.

## Conclusion

We have demonstrated that the two IAs tested give consistently higher results than LC-MS/MS in the range of oestradiol measurements of interest for establishing menopausal status. IAs should not be used to assess menopausal status in women currently or recently treated with fulvestrant. Improved access to mass spectrometry assays would enhance the management of patients with ER-positive breast cancer.

## Supplementary information

Supplemental figure 1

Supplemental figure 2
