# Supplementary figures and images for: Oestradiol measurement during fulvestrant treatment for breast cancer
Source: Br J Cancer. 2019 Jan 25;120(4):404–6. doi: 10.1038/s41416-019-0378-9 (PMC6461991; doi:10.1038/s41416-019-0378-9)

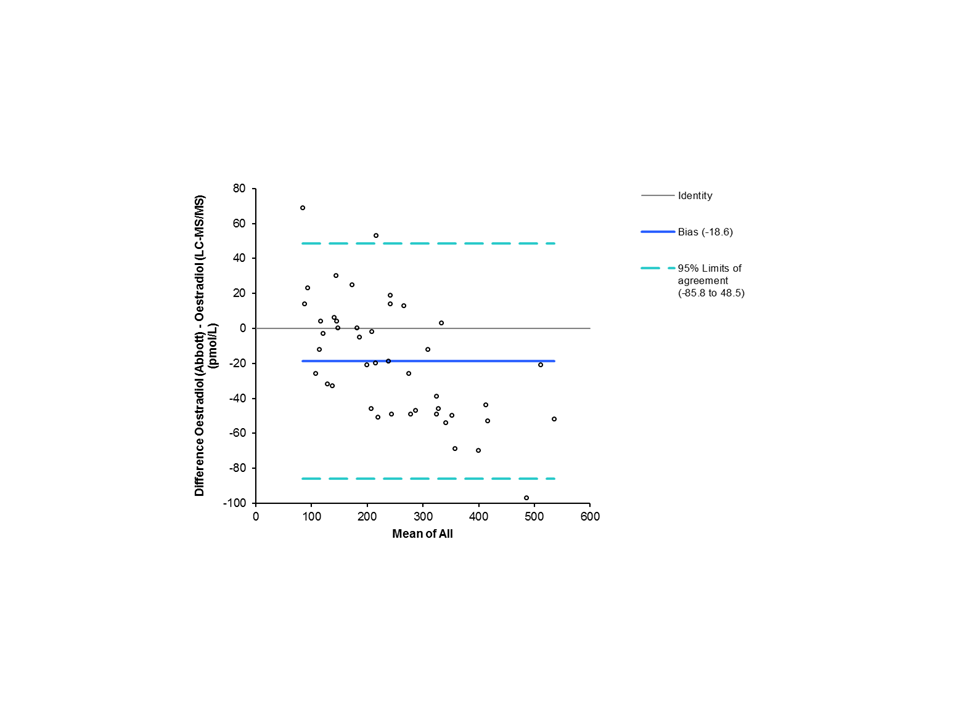

Supplement: Supplementary file 1 — Supplemental figure 1 [file 41416_2019_378_MOESM1_ESM.tif]

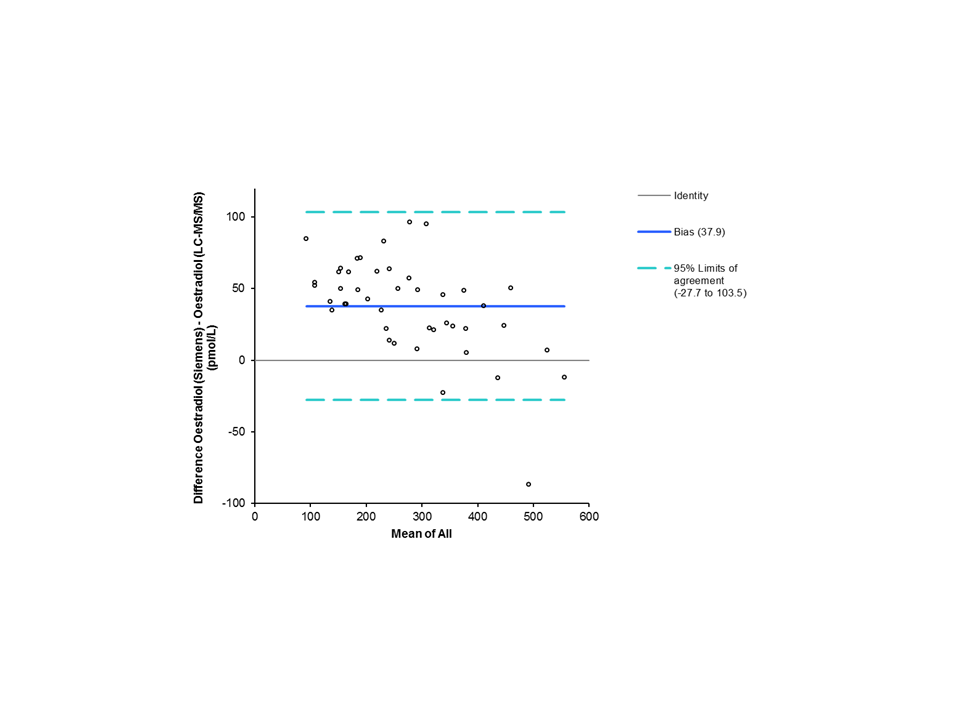

Supplement: Supplementary file 2 — Supplemental figure 2 [file 41416_2019_378_MOESM2_ESM.tif]
